# Simultaneous patellar tendon and anterior cruciate ligament rupture: a systematic review, meta-analysis and algorithmic approach

**DOI:** 10.1007/s00402-024-05676-w

**Published:** 2024-12-18

**Authors:** Petros Ismailidis, Georgios Neopoulos, Christian Egloff, Annegret Mündermann, Florian S. Halbeisen, Corina Nüesch, Christian Appenzeller-Herzog, Sebastian A. Müller

**Affiliations:** 1 Department of Orthopaedics and Traumatology, University Hospital Basel, Spitalstrasse 21, 4031 Basel, Switzerland; 2 Faculty of Medicine, University of Basel, 4031 Basel, Switzerland; 3 Department of Clinical Research, University of Basel, 4031 Basel, Switzerland; 4 University Medical Library, University of Basel, 4051 Basel, Switzerland; 5 Department of Biomedical Engineering, University of Basel, 4031 Basel, Switzerland; 6 Department of Spine Surgery, University Hospital Basel, 4031 Basel, Switzerland; 7 Department of Orthopaedic Surgery and Traumatology, Kantonsspital Baselland (Bruderholz, Liestal, Laufen), 4101 Bruderholz, Switzerland; 8 Surgical Outcome Research Center, University Hospital Basel and University of Basel, Basel, Switzerland; 9 Lower Extremity, Schulthess Klinik, Zurich, Switzerland; 10 Orthopaedic surgery, Leonardo Ortho, Münchenstein, Switzerland

**Keywords:** Patellar tendon, Anterior cruciate ligament, Combined knee injury, Concomitant knee injuries

## Abstract

**Introduction:**

Isolated patellar tendon (PT) or anterior cruciate ligament (ACL) ruptures are common injuries, yet the co-occurrence of both presents a rare challenge for clinicians. The objectives of the study are to document diagnostic and therapeutic approaches, outcomes, and complications of combined PT and ACL injuries and to develop an algorithm to guide clinicians in decision-making.

**Methods:**

The systematic review und metanalysis was conducted according to the PRISMA guidelines. Studies reporting on simultaneous PT and ACL ruptures were included. Meta-analysis was performed to compare different diagnostic modalities and treatment strategies.

**Results:**

Thirty-six studies reporting on 56 Patients were included. 88% of patients had a concomitant injury (apart from PT and ACL) to the ipsilateral knee. Part of the diagnosis was missed in 23% of the cases. Performance of MRI significantly reduced the risk of missing a part of the injury (5% with MRI vs 69% without MRI, *p* < 0.001). Surgical treatment was used only for the PT in 21% of the cases and for the PT and ACL in 77% of the cases (48% one-stage, 52% two-stage surgical treatment).

**Conclusion:**

Combined ACL and PT rupture is rare, and recognizing its full extent is crucial for successful management. Performing an MRI in PT rupture from high-energy trauma and diagnostic arthroscopy/arthrotomy when MRI is not done is essential. PT ruptures should be treated surgically. For ACL rupture, conservative and operative treatment, one- or two-stage surgery are possible based on the patient's profile and concomitant injuries. Based on the limited available literature, this systematic review provides a diagnostic and therapeutic algorithm to assist in clinical decision-making.

**Supplementary Information:**

The online version contains supplementary material available at 10.1007/s00402-024-05676-w.

## Introduction

Isolated patella tendon (PT) or anterior cruciate ligament (ACL) ruptures are common injuries [13, 18, 52]. The management of these injuries is well described in the literature. However, the combination of these two injuries is a rare entity and the literature is mostly limited to case reports. When confronted with this combination of serious injuries, the clinician is faced with several challenges regarding the diagnosis, acute treatment, and rehabilitation.

The first challenge is to correctly diagnose this rare injury. McKinney et al. [41] performed magnetic resonance imaging (MRI) in patients with PT rupture and reported a high incidence of concomitant injuries of the ipsilateral knee, especially to the ACL. Therefore, using the most accurate diagnostic instruments and not overlooking any part of the diagnosis is the first challenging step, before establishing a treatment plan.

Appropriate treatment—surgical or conservative—and timing of surgery are the next major challenges. In general, PT ruptures are treated surgically as soon as possible in order to reconstruct the extensor mechanism, avoid quadriceps muscle atrophy, contractions, and scar formations [19, 63]. Regarding ACL, both surgical and conservative treatment are possible depending on the patient profile [17]. Choosing the surgical treatment for both raises the question of timing [12]. Two-stage surgery could reduce the time between injury and surgical management and minimize the risk of knee stiffness [7, 42]. One-stage surgery, on the other hand, has the advantage of requiring only one intervention, a shorter total period of rehabilitation, and a faster return to previous activity [14, 15, 23, 28].

Postoperatively, the rehabilitation regimen used, especially when choosing a one-stage surgery, is a major challenge because the most commonly used rehabilitation regimens for PT and ACL are contradictory. For instance, after PT surgery, the knee motion is restricted to avoid stressing the PT reconstruction, whereas after ACL injury, early attainment of full range of motion (ROM) is encouraged to avoid postoperative stiffness [58].

Because these combined injuries are rare and always present in an acute setting, decisions must often be made under pressure. An individualized treatment plan based on the injury and the patient profile is required. Since the literature on this topic consists mostly of case reports and small case series, and a comprehensive overview of the literature for guidance in decision making is missing, it is difficult to make the best, evidence-based decision. Therefore, a diagnostic and therapeutic algorithm would be very helpful for clinicians to develop their treatment strategy and could lead to clear and successful decisions. Therefore, the primary objective of this systematic review and meta-analysis was to report the diagnostic and therapeutic approaches, outcomes and complications described in the literature. The secondary objective was to develop a diagnostic and therapeutic algorithm specifically designed for this combination injury.

## Materials and methods

This study was performed according to the PRISMA guidelines [45] for systematic reviews and meta-analysis. A systematic literature search was performed in Embase (via embase.com), Scopus (via scopus.com), Medline (via Ovid) and SportDiscus (via EBSCOhost) from inception to December 4, 2019, and updated on December 7, 2022. References and citing literature from records included from the primary database search were collected by backward and forward citation tracking from TheLens (via citationchaser) and Scopus and screened for eligibility as described below. The protocol was registered in PROSPERO on April 28, 2020 (registration number CRD42020161853). The detailed search strategy is available as Supplementary Material 1. All retrieved references were exported to EndNote 20 (Clarivate, USA) and deduplicated using the Bramer method [6].

Inclusion criteria were human-based studies reporting on simultaneous rupture of the PT and the ACL. Conference abstracts were not included. No further restrictions were placed on study design, publication date, surgical approach, and sex. Exclusion criteria were the reporting of a knee dislocation (because knee dislocations have a different injury mechanism, risks and treatment concept than ACL and PT injuries [34]), patient age younger than 15 years (because injuries in patients with open physis have a different treatment concept than adults [4]) and publication language other than English, German, French, Spanish, Turkish or Greek (to ensure that we have a member in the research team being a proficient user of the language).

The titles and abstracts of the initially retrieved references were screened for potential inclusion. Studies that potentially met the inclusion criteria were included in the full-text screening, which was performed by two authors (PI and SM). A third author was consulted (GN) in case of disagreement on fulfillment of the eligibility criteria. References and citing literature from records included from the primary database search were collected by backward and forward citation tracking from TheLens (via citationchaser [25]) and Scopus and screened for eligibility as described above.

### Quality assessment

The Joanna Briggs Institute (JBI) critical appraisal tool for case reports and case series [41] was used to assess the quality of the included studies, as all of them were case reports or case series. The tool consists of 8 questions for case reports and 10 questions for case series. Each question has four possible responses: “yes”, “no”, “unclear”, or “not applicable”. Answering “no” to any of the questions negatively affects the overall quality of the case report or case series.

### Data extraction

Data from the full texts were extracted and entered into a standardized form by two reviewers (PI and SM). Any discrepancies were resolved by consultation with a third reviewer (GN). Extracted information on study characteristics included the authors and year of publication, country in which the study was conducted, type of study, number of patients, and language in which the study was written. In addition, information on patient characteristics (age, sex, health status, pre-injury knee-specific, activity level with Tegner score if available), mechanism of injury, diagnostic methods, anatomical location of the rupture of the PT (proximal, middle third, distal) and the ACL (proximal, middle third, distal), and accompanying injuries of the ipsilateral and contralateral knee were documented. Finally, information on the part of whether the diagnosis was missed before entering the operating room (in case of missed diagnosis), the treatment, the rehabilitation protocol, the results, the complications and the additional surgeries (if necessary) were documented.

### Outcomes

The main outcomes of this systematic review and meta-analysis were, first, the incidence of missed diagnosis of the combined PT and ACL ruptures and the association of this incidence with the diagnostic tests used; second, the incidence and type of concomitant knee injuries other than the PT and ACL; third the complication rates and outcomes of both conservative and surgical treatment of the ACL rupture; and last, the incidence, complication rates, and outcomes of one-stage and two-stage surgical treatment of both PT and ACL.

Secondary outcomes were the anatomical location of PT rupture and its correlation with the mechanism of injury; the surgical technique (arthroscopic or open) in one-stage surgical treatment of the PT and ACL; and the rehabilitation regimens regarding ROM restrictions, weight bearing, and the incidence and timing of return to sport.

### Statistical analysis

Continuous variables were described as means and standard deviations or medians and ranges when possible. Categorical variables were reported as absolute and relative frequencies.

### Meta-analysis

A meta-analysis was performed to provide a comprehensive comparison between one-stage surgery and two-stage surgery, as well as between missed diagnosis and non-missed diagnosis in terms of patient-reported outcomes (PROMs) and complications. We performed both logistic and linear mixed effects modeling to evaluate the relationships between our predictors and the response variables. For binary outcomes (complications, return to sports, and MRI), logistic mixed-effects models were used, whereas continuous outcomes (postoperative vs. preoperative Lysholm, IKDC) were analyzed using a linear mixed-effects model. Both models included random intercepts to account for the non-independence of measures within a study. All statistical analyses were performed using the R statistical software (Version 4.2.2, The R Foundation for Statistical Computing, Vienna, Austria).

## Results

### Study selection and methodological assessment

**Fig. 1:**
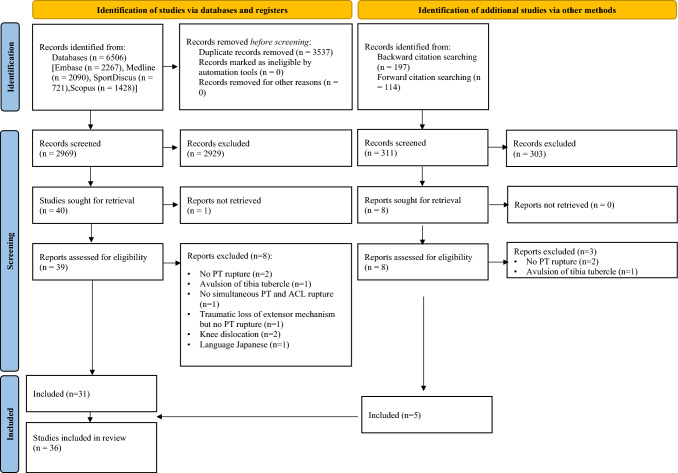
Flow-chart of the study selection process according to the PRISMA 2020 statement: an updated guideline for reporting systematic reviews – available as Additional Material 1

2969 publications were identified from the initial literature search after removing the duplicates. 311 were identified after citation and reference chasing. After the screening process, 36 publications met the eligibility criteria. The detailed overview of the screening process is presented as a PRISMA flowchart (Fig. 1) [45]. Most included studies were case reports (27 studies), four were case series, three were case reports with literature review and two were case series with literature review. The results of the assessment of the methodological quality of all included studies are presented in Supplementary Material 2.

### Extracted information

A total number of 56 patients (46 men and 10 women, median age 30 years, range 15–62 years) were evaluated. Median follow-up was 12 months (range 2–72 months). An overview of the included studies with extracted information on number of patients, study type, patient demographics, activity level and follow-up time is presented in Table 1.

**Table 1:** Overview of the included studies including extracted information regarding number of patients, study type, patient demographics, activity level (Tegner Score*), mechanism of injury (namely*) and follow-up time

References	Number of Patients	Study type	Age (years)	Gender	Activity level (Tegner Score*)	Mechanism of injury (namely*)	Follow-up, months
Baker [3]	1	Case report	24	m	n/a	Basketball (ER stress in a flexed knee)	n/a
Brunkhorst [7]	1	Case report	27	m	n/a	Soccer (R stress)	n/a
Chiang [10]	1	Case report	30	m	n/a	Basketball (R stress)	12
Chiba [11]	1	Case report	36	m	n/a	Baseball (unclear stress on a flexed knee)	31
Chow [12]	1	Case report	23	m	n/a	Basketball (while jumping, no direct impact)	21
Costa-Paz [14]	3	Case series^#^	31	m	n/a	Motorcycle acc	48
			31	m	Recreational soccer player	Soccer (R stress, impact)	36
			50	m	nm	Bicycle acc	24
Cucchi [15]	2	Case report^#^	35	m	n/a	Ski (IR stress)	12
			44	m	n/a	Ski (R stress)	12
Futch [23]	1	Case report	19	m	n/a	Football (deceleration with fixed foot and flexed knee)	n/a
Kim [30]	1	Case report	32	m	n/a	Baseball (fixed foot, valgus stress on flexed knee)	12
Koukoulias [31]	1	Case report	47	m	n/a	Unclear	36
McCormack [40]	1	Case report	26	m	Pro football player	Football (R stress with fixed foot)	n/a
Rae [50]	1	Case report	25	w	n/a	Trampoline (forced flexion on extended knee)	24
Levakos [33]	6	Case series	36	m	Recreational soccer player	Soccer (landing)	18
			15	w	School athlete	Long jump (landing)	72
			33	m	n/a	Ski (landing)	72
			20	m	College football athlete	Football (R stress on a fixed foot)	n/a
			23	m	n/a	Motorcycle acc	12
			23	m	Pro football player	Football (R stress on a fixed foot)	6
Mariani [37]	3	Case series	45	M	Competitive skier	Ski (Valgus and ER stress)	7.5
			19	w	Pro modern dancer	Trampoline (Valgus and ER stress	9
			18	w	n/a	Wake board (Valgus and ER stress)	12
Shillington [55]	1	Case report	23	m	State level rugby leave	Rugby (Single leg stance and impact with 3 defenders or valgus and hyperflexion stress)	7.5
Tsarouhas [56]	1	Case report	38	m	Recreational martial arts	Martial arts (Impact and Valgus stress)	11
Wissman [61]	1	Case report	36	w	n/a	nm (fall from height and landed on both feet)	n/a
Gülabi [24]	1	Case report	30	m	Recreational soccer player	Soccer (deceleration with foot fixed and knee flexed)	12
Capogna [9]	6	Case series	40	m	n/a	Bicycle struck by motorcycle	2 (lost to follow-up)
			57	m	n/a	Struck by a car	3 (lost to follow-up)
			36	m	n/a	Struck by motorcycle	7
			62	m	n/a	nm (jumping to avoid a ball on the street)	4
			20	m	n/a	Trampoline (R stress)	6
			20	w	n/a	Trampoline (while landing)	9
Achkoun [1]	1	Case report	22	m	n/a	Soccer (R stress of lower leg with blocked foot)	6
Schmidt-Wiethoff [53]	1	Case report	28	w	n/a	Ski (ER and valgus stress)	5
Vega [59]	1	Case report	31	m	n/a	Soccer (R stress)	6
Perez [46]	1	Case report	21	m	n/a	Soccer (by landing, without able to describe the exact mechanism)	18
Mathews [39]	1	Case report^#^	41	m	n/a	Motocross (landing on foot with flexed knee)	36
Lobo [36]	1	Case report	30	m	n/a	Traffic acc. (Direct blow on the knee and valgus stress)	6
Ismailidis [29]	1	Case report	40	w	n/a	Ski (R stress, no direct impact)	24
Quinn [49]	3	Case series^#^	20	m	Collegiate football athlete	Football (flexed knee, fixed leg, varus stress)	12
			15	m	School football player	Football (tackled)	12
			17	m	Lacrosse player	Football (ER and valgus stress, direct contact)	15
Boublik [5]	3	Case series	nm	m	National football league player (10)	Football (tackled)	n/a
			nm	m	National football league player (10)	Football (R stress, direct blow)	n/a
			nm	m	National football league player (10)	Football (Non contact injury, no other info)	n/a
Verma [60]	2	Case report	20	m	nm	Traffic acc	18
			34	m	nm (4)	Traffic acc	12
Ansari [2]	1	Case report	37	m	Recreational soccer player	Football (Leg twisted beneath him)	36
Pointinger [48]	1	Case report	25	m	n/a	nm (ER and valgus stress on flexed knee)	3
Perwanger [47]	1	Case report	37	m	n/a	Ski (high velocity, hyperflexion, R stress)	6.5
Xie [62]	1	Case report	46	m	n/a	Traffic acc. (Translation and valgus of proximal tibia with flexed knee and eccentric quadriceps contracture)	12
A.De Rousiers [16]	1	Case report^#^	39	w	Recreational sportswoman (climbing, swimming, running)	Climbing (R stress after hyperextension landing)	18
Selva-Sarzo [54]	1	Case report	28	m	Pro handball player	Handball (IR and valgus after landing)	24
Zhong Li [35]	1	Case report	18	w	n/a	Long jump (sprained knee)	12

*BMI* body mass index (kilogram/meter^2^), *SD* standard deviation, *n/a* Not available, *: If available, #: with a review of the literature, *R* rotational, *ER* external rotation, *IR* internal rotation, *acc* accident

### Mechanism of injury

Regarding the mechanism of injury, 77% (43 of 56) of patients had a sports-related injury, while 18% (10 of 56) had an injury resulting from a traffic accident. 41% of patients (23 of 56) described a rotational stress on the knee (either internal or external) in addition to a varus or valgus stress in a partially flexed knee [1, 3, 5, 7, 9, 10, 14–16, 29, 33, 37, 40, 47, 48, 53, 54]. In addition, 30% of patients (17 of 56) clearly demonstrated rapid deceleration and consequent quadriceps contraction in a flexed knee [3, 9, 12, 16, 23, 24, 37, 39, 46, 47, 50, 54, 61]. The data collected are shown in Table 1.

### Diagnostic modalities

Regarding the diagnostic modalities used, radiography and clinical examination were performed in all cases, sonography in 4%, and a MRI in 71% (40 of 56) of patients (Table 2) [1, 5, 7, 9–11, 14–16, 23, 24, 30, 33, 35–37, 39, 46, 47, 53, 55, 60–62].

**Table 2:** Detailed information on the diagnosis, the concomitant injuries, the performance of MRI as well as the part of the diagnosis missed clinically and after applying the radiological diagnosis, prior to entering the operating room

References	Number of Patients	MRI	Location of PT rupture	Location of ACL rupture	Concomitant injuries	Missed clinically (namely)	Missed prior to operation (namely)
Baker [3]	1	nm	n/a	Mid-substance	MCL, MM	n/a	n/a
Brunkhorst [7]	1	Yes	n/a	n/a	MCL, LM	No	No
Chiang [10]	1	Yes	Mid-substance	Mid-substance	LM	No	No
Chiba [11]	1	Yes	Proximal	Distal	MCL	No	No
Chow [12]	1	No	Proximal	n/a	MM, LM	Yes (MM, LM)	Yes (MM, LM)
Costa-Paz [14]	3	Yes	Mid-substance	n/a	MCL	No	No
		Yes	Mid-substance	n/a	MCL	Yes (PT)	No
		Yes	n/a	n/a	LM	Yes (ACL, LM)	No
Cucchi [15]	2	Yes	n/a	n/a	MCL, MM	Yes (ACL, MM)	Yes (MM)
		Yes	n/a	n/a	MCL, LCL	No	No
Futch [23]	1	Yes	Mid-substance	Mid-substance	MM, LM	No	No
Kim [30]	1	Yes	Mid-substance	Proximal	MCL, LM	No	No
Koukoulias [31]	1	No	Mid-substance	n/a	MCL, MM,LM, chondral defect medial femoral condyle	Yes (ACL, MM, LM, chondral defect medial femoral condyle)	Yes (ACL, MM, LM, chondral defect medial femoral condyle)
McCormack [40]	1	No	Mid-substance	Proximal	MM, medial and lateral patellar retinaculum	Yes (MM, medial and lateral patellar retinaculum)	Yes (MM, medial and lateral patellar retinaculum)
Rae [50]	1	No	Mid-substance	n/a	MCL	Yes (PT)	Yes (PT)
Levakos [33]	6	No	Mid-substance	n/a	MCL, MM, LM	Yes (PT)	Yes (PT)
		No	Mid-substance	n/a	No	Yes (ACL)	Yes (ACL)
		No	Mid-substance	n/a	MM, LM	Yes (PT, MM, LM)	Yes (PT)
		Yes	Mid-substance	n/a	MCL, LM	No	No
		No	Proximal	n/a	MCL, MM, LM	Yes (ACL, MCL, MM, LM)	Yes (ACL, MCL, MM, LM)
		Yes	Mid-substance	Mid-substance	MCL	No	No
Mariani [37]	3	Yes	Mid-substance	n/a	MCL, MM	No	No
		Yes	Mid-substance	n/a	MCL, MM, LM	No	No
		Yes	nm	n/a	MCL, MM	No	No
Shillington [55]	1	Yes	nm	n/a	MCL, MM	No	No
Tsarouhas [56]	1	No	proximal	n/a	MCL, LM	Yes (ACL, MCL, LM)	Yes (ACL, MCL, LM)
Wissman [61]	1	Yes	Mid-substance	Mid-substance	No	Yes (PT)	No
Gülabi [24]	1	Yes	Mid-substance	Mid-substance	MCL	No	No
Capogna [9]	6	Yes	Distal	n/a	MCL, MM, lateral tibia plateau fracture, partial tear of biceps femoris	No	No
		Yes	Distal	n/a	MCL, MM, PCL	No	No
		No	Distal	n/a	MCL, posterolateral tibia plateau fracture	No	No
		Yes	Distal	n/a	MCL, MM, LM, PCL, posterolateral tibia plateau fracture, avulsion of iliotibial band	No	No
		Yes	Distal	n/a	MCL	No	No
		Nm	Distal	n/a	MCL	No	No
Achkoun [1]	1	Yes	nm	n/a	No	No	No
Schmidt-Wiethoff [53]	1	Yes	Mid-substance	n/a	MCL, MM	No	No
Vega [59]	1	Yes	Mid-substance	n/a	No	No	No
Perez [46]	1	Yes	Proximal	n/a	No	No	No
Mathews [39]	1	Yes	nm	n/a	MM	Yes (PT, MM)	No
Lobo [36]	1	Yes	Mid-substance	n/a	MCL	No	No
Ismailidis [29]	1	No	Mid-substance	Proximal	MM, LM, tibia plateau fracture	Yes (ACL, PT, MM, LM)	Yes (ACL, MM, LM)
Quinn [49]	3	Yes	Proximal	Proximal	MCL	No	No
		Yes	n/a	n/a	MCL, MM, LM	No	No
		Yes	Distal	n/a	MCL, LM, medial capsule and retinaculum	Yes (ACL, LM)	No
Boublik [5]	3	Yes	n/a	n/a	LM	n/a	No
		Yes	n/a	n/a	Chondral defect in trochlea	n/a	No
		Yes	n/a	n/a	No	n/a	No
Verma [60]	2	Yes	Proximal	n/a	MCL, LM	Yes (ACL, PT, MCL, LM	No
		Yes	Proximal	n/a	MCL, LM	Yes (ACL, PT, MCL, LM)	No
Ansari [2]	1	No	n/a	n/a	No	Yes (ACL)	Yes (ACL)
Pointinger [48]	1	No	n/a	n/a	MCL, LM, PCL, pes anserinus lesion, chondral fragment lateral femoral condyle	Yes (PT, pes anserinus lesion)	No
Perwanger [47]	1	Yes	Mid-substance	Mid-substance	LM	No	No
Xie [62]	1	Yes	n/a	n/a	MCL, tibia plateau fracture	Yes (MCL)	No
De Rousiers [16]	1	Yes	n/a	n/a	MCL	Yes (ACL, PT, MCL)	No
Selva-Sarzo [54]	1	nm	n/a	n/a	MCL, MM	n/a	No
Zhong Li [35]	1	Yes	n/a	n/a	MCL, MM, LM	Yes (LM, MM)	Yes (MM)

*MRI* magnetic resonance imaging, *PT* patellar tendon, *ACL* anterior cruciate ligament, *PCL* posterior cruciate ligament, *MCL* medial collateral ligament, *LCL* lateral collateral ligament, *MM* medial meniscus, *LM* lateral meniscus, *n/a* not available

### Localization of the PT and ACL rupture

The most frequently reported rupture was a mid-substance rupture of the PT (59% of patients) [10, 14, 23, 24, 29–31, 33, 36, 37, 40, 47, 50, 53, 61], whereas a distal PT rupture occured in 19% of cases, where the location of the rupture was reported. It should be noted, however, that in a considerable proportion of cases (34% of patients) there was no information on the location of the rupture. For ACL rupture, the anatomical location of the rupture was not reported in most cases, with 79% of cases lacking information. 58% of cases reporting the location of the ACL rupture, presented with a mid-substance and 33% with a proximal ACL rupture. (Table 2).

### Accompanying injuries

88% of patients had a concomitant injury (apart from PT and ACL) to the ipsilateral knee, with 68% having a medial collateral ligament (MCL) rupture, 39% a medial meniscus lesion, and 41% a lateral meniscus lesion (Table 2).

### Missed diagnosis

The diagnosis of the PT, ACL rupture, or concomitant injuries was clinically missed in 20% (11 out of 56), 21% (12 of 56), and 30% (17 of 56) of patients, respectively, at the initial examination. PT was missed in 5% (3 of 56), ACL in 11% (6 of 56), and concomitant injuries in 14% (8 of 56) of patients prior to admission to the operating room, even after radiologic diagnosis. Overall, patients entered the operating room with a part of the diagnosis missing in 23% (13 out of 56) cases. Except for two patients in whom a medial meniscus injury was overlooked even after performing MRI [15, 35], all other patients in whom part of the diagnosis was missed did not undergo an MRI [2, 12, 29, 31, 33, 40, 49, 55]. Hence all combined injuries of PT and ACL were depicted on the MRI performed. Detailed information about the diagnosis and the missed part of the diagnosis is shown in Table 2.

### Treatment approach: surgical or conservative

Regarding the treatment approach, surgery was chosen in 77% (43 of 56) of cases for both PT and ACL [1, 2, 5, 7, 9–11, 14, 15, 23, 24, 29–31, 33, 35–37, 39, 40, 46, 47, 53–56, 60], with arthroscopic reconstruction of the ACL accounting for 72% of these cases. Conversely, in 21% (12 of 56) of cases, surgical treatment was chosen for the PT and conservative treatment for the ACL rupture [3, 9, 12, 16, 33, 48, 50, 62]. Only one patient was treated surgically for the ACL and conservatively for the PT rupture [61]. As for the concomitant lesions, the MCL and medial meniscus were treated surgically in the majority of patients (55% of them with MCL and 68% of them with medial meniscus rupture, respectively). Suture and partial meniscectomy were chosen for lateral meniscus lesions in approximately equal numbers, 43% and 35%, respectively (Table 4). Detailed information on the treatment strategies of the individual studies regarding the PT, ACL, and concomitant injuries can be found in Table 3.

**Table 3:** Treatment strategies of the individual studies regarding the patellar tendon, anterior cruciate ligaments and concomitant injuries

References	Number of patients	Management	Surgical timing (interval in weeks if two-stage)	Treatment ACL (technique)	Treatment PT	Treatment concomitant injuries
Baker [3]	1	PT op ACL cons	nr	nr	Suture + wire	MCL: suture MM: partial meniscectomy
Brunkhorst [7]	1	PT, ACL op	Two-stage (nm)	Arthroscopic (n/a)	Suture + transosseus suture	MCL: n/a LM: suture
Chiang [10]	1	PT, ACL op	One-stage	Arthroscopic (autograft ipsilateral, hamstrings)	Suture + transosseus suture	LM: partial removal and fixation of the rest with meniscal arrows
Chiba [11]	1	PT, ACL op	Two-stage (28)	Arthroscopic (not clear, semitendinosus)	Suture + transosseus suture	MCL: suture
Chow [12]	1	PT op ACL cons	nr	nr	Suture + wire	MM: suture LM: partial meniscectomy
Costa-Paz [14]	3	PT, ACL op	Two-stage (5)	Arthroscopic (autograft contralateral, BPTB)	Suture	MCL: suture
		PT, ACL op	One-stage	Arthroscopic (not clear, quadriceps)	Suture	MCL: suture
		PT, ACL op	One-stage	Arthroscopic (autograft ipsilateral, gracilis and semitendinosus)	suture	LM: partial meniscectomy
Cucchi [15]	2	PT, ACL op	One-stage	Arthroscopic (autograft ipsilateral, gracilis and semitendinosus)	Suture + wire	MCL: suture MM: suture
		PT, ACL op	One-stage	Arthroscopic (autograft ipsilateral, gracilis and semitendinosus)	Suture + wire	
Futch [23]	1	PT, ACL op	One-stage	Open (allograft BPTB)	Suture + allograft PT	MM: suture
Kim [30]	1	PT, ACL op	One-stage	Arthroscopic (allograft, achilles tendon)	Suture + transosseus suture	MCL: cons LM: suture
Koukoulias [31]	1	PT, ACL op	Two-stage (24)	nm (autograft ipsilateral, hamstrings)	Suture + wire	MCL: cons MM: cons LM: partial meniscectomy
McCormack [40]	1	PT, ACL op	Two-stage (12)	Arthroscopic (autograft ipsilateral, gracilis and semitendinosus)	Suture + transosseus suture	MM: suture LM: partial meniscectomy
Rae [50]	1	PT op ACL cons	nr	nr	Suture	MCL: suture
Levakos [33]	6	PT, ACL op	One-stage	n/a (synthetic)	Suture	MCL: suture MM: suture LM: suture
		PT, ACL op	Two-stage (156)	n/a (autograft ipsilateral, iliotibial band)	Suture	n/a
		PT, ACL op	One-stage	n/a (autograft ipsilateral, semitendinosus)	Suture	MM: suture LM: partial meniscectomy
		PT op ACL cons	nr	nr	Suture	MCL: suture LM: cons
		PT op ACL cons	nr	nr	Suture	Missed
		PT, ACL op	Two-stage (12)	n/a (allograft, n/a)	Suture	n/a
Mariani [37]	3	PT, ACL op	Two-stage (6)	Arthroscopic (autograft ipsilateral, hamstrings)	Suture	MCL: suture MM: suture
		PT, ACL op	Two-stage (13)	nm (autograft ipsilateral, hamstrings)	Suture + wire	MCL: suture MM: suture LM: suture
		PT, ACL op	Two-stage (10)	Arthroscopic (autograft ipsilateral, hamstrings)	Suture	MCL: suture MM: suture
Shillington [55]	1	PT, ACL op	Two-stage (14)	Arthroscopic (autograft contralateral, hamstrings)	Suture + transosseus suture	MCL: suture MM: cons
Tsarouhas [56]	1	PT, ACL op	Two-stage (12)	Arthroscopic (autograft ipsilateral, hamstrings)	Suture + transosseus suture	MCL: cons LM: partial meniscectomy
Wissman [61]	1	PT cons ACL op	nr	Arthroscopic (autograft ipsilateral, hamstrings)	nr	nr
Gülabi [24]	1	PT, ACL op	One-stage	Arthroscopic (autograft contralateral, gracilis and semitendinosus)	Suture + transosseus suture + semitendinosus and gracilis augmentation in figure of 8 style	MCL: cons
Capogna [9]	6	PT op ACL cons	nr	nr	suture anchor fixation	MCL: cons Tibia fracture: cons
		PT op ACL cons	nr	nr	suture anchor fixation	MCL: suture MM: suture
		PT op ACL cons	nr	nr	nm	MCL: suture Tibia fracture: ORIF Femur fracture: cons
		PT op ACL cons	nr	nr	Suture anchor fixation	MCL: suture MM: partial meniscectomy and suture LM: suture PCL: cons Tibia fracture: cons Iliotibial band: suture anchor
		PT, ACL op	Two-stage (12)	Arthroscopic (autograft ipsilateral, BPTB)	Suture anchor fixation	MCL: suture
		PT, ACL op	Two-stage (16)	Arthroscopic (autograft ipsilateral, hamstrings)	Suture anchor fixation	MCL: cons
Achkoun [1]	1	PT, ACL op	Two-stage (n/a)	Arthroscopic (autograft ipsilateral, gracilis and semitendinosus)	Suture + wire	nr
Schmidt-Wiethoff [53]	1	PT, ACL op	One-stage	Arthroscopic (autograft ipsilateral, gracilis)	Suture + wire	MCL: suture MM: suture
Vega [59]	1	PT, ACL op	One-stage	Arthroscopic (autograft ipsilateral, gracilis and semitendinosus)	Suture + semitendinosus autograft augmentation (contralateral)	nr
Perez [46]	1	PT, ACL op	One-stage	Arthroscopic (autograft ipsilateral, gracilis and semitendinosus)	Anchor + semitendinosus and gracillis autograft augmentation (contralateral)	nr
Mathews [39]	1	PT, ACL op	One-stage	Arthroscopic (autograft contralateral, hamstrings)	Suture	MM: suture
Lobo [36]	1	PT, ACL op	Two-stage (6)	Arthroscopic (autograft ipsilateral, hamstrings)	Suture + transosseus suture	MCL: cons
Ismailidis [29]	1	PT, ACL op	One-stage	Open (refixation, direct repair)	Suture + wire	MM: suture LM: suture Tibia fracture: ORIF
Quinn [49]	3	PT, ACL op	Two-stage (12)	Arthroscopic (allograft, BPTB)	Suture + transosseus suture	MCL: suture
		PT, ACL op	Two-stage (12)	Arthroscopic (autograft ipsilateral, quadriceps)	Suture	MCL: internal brace MM: suture LM: cons
		PT, ACL op	Two-stage (24)	Arthroscopic (allograft, semitendinosus)	Suture + transosseus suture	MCL: suture LM: partial meniscectomy
Boublik [5]	3	PT, ACL op	One-stage	nm (autograft, contralateral, BPTB)	n/a	LM: nm
		PT, ACL op	One-stage	n/a (allograft, n/a)	n/a	nr
		PT, ACL op	Two-stage (4)	n/a	n/a	nr
Verma [60]	2	PT, ACL op	One-stage	Arthroscopic (autograft contralateral, hamstrings)	Suture + transosseus suture	MCL: autograft LM: partial meniscectomy
		PT, ACL op	One-stage	Arthroscopic (autograft contralateral, hamstrings)	Suture + transosseus suture	MCL: autograft LM: partial meniscectomy
Ansari [2]	1	PT, ACL op	Two-stage (104)	Arthroscopic (autograft ipsilateral, semitendinosus)	Suture	nr
Pointinger [48]	1	PT op ACL cons	Two-stage (ACL plastic in the future)	nr	Suture	MCL: suture LM: partial meniscectomy PCL: resected initial Chondral fragment: removed Retinaculum: suture
Perwanger [47]	1	PT, ACL op	Two-stage (14)	Arthroscopic (autograft ipsilateral, semitendinosus)	Suture + transosseus suture	LM: suture ALL: reconstruction
Xie [62]	1	PT op ACL cons	nr	nr	Suture + wire	MCL: suture Tibia fracture: cons
De Rousiers [16]	1	PT op ACL cons	nr	nr	Suture	MCL: reinsertion distal
Selva-Sarzo [54]	1	PT, ACL op	One-stage	Arthroscopic (autograft ipsilateral, gracilis and semitendinosus)	Suture + wire + semitendinosus and gracilis autograft augmentation (contralateral)	MCL: cons MM: partial meniscectomy
Zhong Li [35]	1	PT, ACL op	One-stage	Arthroscopic (autograft ipsilateral, gracilis and peroneus longus)	Suture	MCL: autograft (semitendinosus) MM: suture LM: suture

*op.* operation, *cons.* conservative, *nr* non-relevant, *PT* patellar tendon, *ACL* anterior cruciate ligament, *PCL* posterior cruciate ligament, *MCL* medial collateral ligament, *LCL* lateral collateral ligament, *MM* medial meniscus, *LM* lateral meniscus, *BPTB* bone-patellar tendon-bone, *ORIF* open reduction, internal fixation, *ALL* anterolateral ligament, *n/a* not available

### Timing of surgery: one versus two-stage

Regarding the timing of surgery, when both the PT and ACL are treated surgically, there is an almost equal distribution of studies favoring one-stage (48%, 21 of 44 patients) and two-stage surgery (52%, 23 of 44 patients) approaches. The median interval for two-stage surgery was 12 weeks (range 4–104 weeks). Table 3 shows the reported treatment strategies.

### Rehabilitation protocols

Depending on the treatment strategy, different rehabilitation protocols were followed.

One-stage treatment: A cast was used in 95% of cases. All studies reporting on weight-bearing used partial or no weight-bearing for a median duration of 6 weeks (range 2–8 weeks), whereas 38% did not report on weight-bearing. ROM restrictions were used in all patients for whom ROM was reported, while no information was available in 19%.

Two-stage treatment or surgical treatment of PT alone: A cast was used in 60% of cases. 40% of patients were reported to have followed a partial or no weight bearing for a median of 5 weeks (range 2–6 weeks), while 53% lacked information on weight bearing. Of the studies that reported ROM regimens, ROM restrictions were used in 85% of patients. In 40% of the patients, no information on ROM was provided.

### Complications and additional procedures

Regarding complications, 20% of the patients (11 of 56) had a documented complication. The most common complications were arthrofibrosis and patella baja, each observed in 7% of patients, while infection (either deep or superficial) and complex regional pain syndrome (CRPS) was observed in 4% and 2% of patients, respectively. A total of 14 additional procedures were required in 12 patients (21%). Removal of the wire used to secure the PT suture was performed in six cases, arthroscopy in five cases (2 for synovectomy, 1 for debridement of scar tissue and resection of suture and 2 for removal of the wire and diagnostic purposes), and revision for infection in two cases. Arthrolysis, fasciocutaneous flap coverage, closed manipulation, and arthroscopy with debridement of the scar tissue and resection of suture material were each performed in one case each.

### Statistical analysis and meta-analysis

When comparing patients with and without missed diagnoses prior to entering the operating room, there were some interesting findings.

#### Importance of MRI

Performing MRI as part of the diagnostic workup is clearly the key to not missing part of the diagnosis. In the group where MRI was performed, only 5% had part of the diagnosis missed, while in the group where MRI was not performed, in 69% of cases part of the diagnosis was missed (*p* < 0.001, Table 4). In the MRI group, only minor injuries (meniscal lesions) were missed.

**Table 4:** Statistical analysis of the group receiving an MRI and the group not receiving an MRI as part of the diagnostics, with regards to missing a part of the diagnosis and presenting complications

Group	Diagnosis missed	Complications
With mri N = 40	5% (2/40)	6/40 (15.4%)
No mri N = 16	69% (11/16)	5/16 (31.2%)
*P* Value Test performed	< 0.001	0.343

#### “Missed diagnosis vs. non-missed diagnosis”

Interestingly the “missed diagnosis” group had only slightly more complications than the “non-missed diagnosis” group (23% vs. 19%, *p* = 0.676), with markedly different groups sizes however. The clinical scores of the two groups were also comparable. The statistical results and clinical scores are shown in Table 5.

**Table 5:** Statistical analysis comparing the patients, where a part of the diagnosis was missed prior to entering the operating room (“missed diagnosis) to those where no part of the diagnosis was missed (“non-missed”)

Group	Complications	Return to sports (patients returning to sports/total patients with reported return to sports as outcome/no data reported)	IKDC score	Lysholm score
Missed diagnosis N = 13	23% (3/13)	44% (6/8/5)	78.3 (SD 16.5) (Reported in 2/13)	89.5 (SD 6.4) (Reported in 2/13)
Non missed diagnosis N = 43	19% (8/43)	40% (17/20/23)	85.91 (SD 11.4) (Reported in 12/43)	Lysholm: 88.4 (SD 11.9) (Reported in 17/43)
*P* value	0.676	0.567	0.129	0.394

*IKDC* International Knee Documentation Committee, *SD* Standard Deviation

#### One-stage versus two-stage

When comparing patients who underwent one-stage surgery with those who underwent two-stage surgery, the complication rate was higher in the one-stage group, however statistically not significant (9% vs. 19%, *p* = 0.88). Conversely, 12/14 patients (86%) return to sport after “one-stage” surgery, while 7/9 (78%) after “two-stage” surgery, without a statistically significant difference between them (*p* = 0.88). The statistical results as well as the clinical scores are presented in Table 6. It is important to mention than the data was only available in a portion of the studies, as indicated in Table 6

**Table 6:** Statistical analysis of patients receiving a surgical treatment of both the ACL and PT, separated on the group of one stage and two stage treatment

Group	Complications	Return to sports (patients returning to sports/total patients with reported return to sports as outcome/no data given)	Lysholm	IKDC
One-stage N = 21	19% (4/21)	86% (12/14/7)	86.30 (SD 14.67)	83.23 (SD 15.62)
Two-stage N = 23	9% (2/23)	78% (7/9/14)	92.5 (SD 4.41)	87.43 (SD 5.84)
*P* value	*P* = 0.88	*P* = 0.88	0.376	0.939

*IKDC* International knee documentation committee, *SD* Standard deviation

## Discussion

The primary aim of this study was to report the diagnostic and therapeutic approaches, outcomes and complications of combined PT and ACL injury. The secondary aim was to develop a diagnostic and therapeutic algorithm.

### Missed diagnosis

The most important finding of this study is that part of the diagnosis was missed in 23% of cases. This percentage drops dramatically if an MRI is performed no case missing a tear of the ACL nor the PT. Only a meniscal tear was missed on the MRI in 5% of the cases, not substantially changing the treatment approach. As shown in this review, the management of such complicated injuries requires an in-depth examination of the injuries, and therefore only a complete diagnosis allows for a structured treatment plan. Therefore, establishing the correct diagnosis prior to entering the operating room seems to be the key to successful treatment. This is particularly important because many surgeons still consider a rupture of the PT to be a “clinical diagnosis”, supported at most by sonography. Therefore, as shown in this review, MRI is often not performed and part of the diagnosis could be missed. Interestingly, in patients with missed diagnosis, complications were only slightly higher and clinical outcomes were not worse. However, it is important to note that the groups sizes were markedly different and the *p* value (*p* = 0.676) indicates that this difference is not statistically significant. This finding should be interpreted with caution as the lack of statistical significance does not confirm the absence of a difference but rather that the study might be underpowered to detect a difference. Moreover, this result is probably related to the small number of reported cases and should not be used as an argument to avoid acquiring a full diagnosis prior to the operation.

The available literature supports the findings of this review that associated injuries are very common in cases of PT rupture. McKinney et al. [41] reported an 18% incidence of ACL rupture detected in routine MRI in patients with already clinically diagnosed PT rupture following a low energy trauma (non-contact sports, walking or activities of daily living, fall from standing and chronic PT ruptures), and 38% incidence after a high energy trauma. While PT can be easily diagnosed clinically and/or with a sonography most of the times, the same does not apply for an ACL rupture and for the rest concomitant injuries (MCL, meniscal injuries). Therefore, an MRI should be performed in all high-energy injuries with PT rupture. In the case of low energy trauma with inconclusive clinical examination or when an MRI is not available, a diagnostic arthroscopy or direct visual examination should be performed during surgical treatment to avoid missing part of the diagnosis.

### Treatment concepts

#### Operative versus conservative treatment

The management of this rare injury is controversial in the available literature. There is a clear consensus for immediate surgical reconstruction of the PT rupture [3, 10, 12, 14, 23, 33, 38, 50], in order to restore the extensor mechanism, avoid any atrophy or contraction of the quadriceps muscle, and also avoid scar formation of the PT. In the setting of the combination injury, our review revealed that a surgical treatment was chosen in 77% of the cases, whereas 21% of cases opted for surgical treatment of the PT rupture and conservative treatment for the ACL rupture.

The indication for surgical treatment of the ACL must be decided individually according to the patient’s profile [17]. If the patient’s profile indicates that surgical treatment of the ACL might not be necessary, one can wait until the completion of PT rehabilitation and then decide on the need for ACL reconstruction according to the current guidelines based on the degree of knee instability and activity level.

In the presence of a repairable meniscal injury (e.g., bucket handle tear), a stable ACL is required, otherwise the risk of meniscal repair failure is unacceptable [51]. Similarly, the ACL should be treated surgically in patients participating in high-level pivot-shift sports [20, 32, 44]. In these cases where surgical treatment of the ACL is indicated, the timing of surgery as well as the methods are controversial.

#### One-stage versus two-stage surgical treatment

In cases of repairable large meniscal lesions, where meniscus repair is indicated, two-stage surgery with a long interval is not a reasonable option due to the high risk of meniscal repair failure in the absence of an ACL [51]. In these cases, one-stage treatment should be favored. In the rest of the cases one or two-stage treatment is an option. Arguments in favor of two-stage surgery are the reduction of the risk of arthrofibrosis and the postoperative ROM deficit observed in one-stage surgery [10, 23]. On the other hand, one-stage surgery offers the obvious advantage of fewer surgeries and shorter rehabilitation time as well as satisfactory outcomes [14, 15, 55]. However, one-stage treatment makes the rehabilitation more difficult because of the conflicting rehabilitation protocols after PT repair (protected, limited, gradually increasing ROM) compared to after ACL reconstruction (early ROM encouraged).

In a systematic review published in 2018, Meheux et al. [42] found a significantly higher complication rate with no significant difference in return to preinjury activity level. Our review is consistent with that review in terms of the complication rates, with 19% reported in the one-stage group and 9% in the two-stage group, however without a statistical significant different. This may be due to the presence of inflammation, swelling, and the ROM deficit at the time of ACL reconstruction, which would also explain arthrofibrosis being the most common complication reported. In summary, both one-stage and two-stage surgeries are reasonable options and can be chosen on an individual basis.

#### Surgical ACL treatment: reconstruction versus repair

Arthroscopic ACL reconstruction with autograft (hamstring or PT) is the gold standard for ACL treatment [20]. However, in the specific setting of combined ACL and PT injuries, additional questions arise. A PT autograft is obviously not an option, and an ipsilateral hamstring autograft, especially in cases of concomitant MCL rupture, would be an additional trauma and further weaken the knee. Therefore, an acute repair of proximal Type I ACL ruptures [57] may be a good option. Such repairs have yielded satisfactory results [8, 26, 43], and although not established for standard ACL treatment, they offer obvious advantages in combined ACL and PT injuries. A repair was only performed in one case of the ones included in this review [29]. An allograft would be an alternative option. The data from our review do not allow for a statistical comparison of repair versus autograft versus allograft reconstruction, as only 5% of the cases treated by one-stage surgery had a repair and 14% had an allograft.

### Rehabilitation regimens

Rehabilitation regimens after PT rupture differ from those after ACL rupture. The ACL requires an early ROM and an accelerated rehabilitation, whereas PT demands a period of immobilization and restricted ROM [19, 29, 38]. This is an important argument for surgeons for choosing the two-stage approach. On the contrary, with the one-stage surgery, encouraging an early postoperative ROM is crucial to prevent arthrofibrosis. A gradual increase in ROM with non-weight bearing for 6 weeks is supported, taking into account the strength of the PT repair. According to two studies included in our review, rehabilitation alone should not preclude a one-stage approach, as a combined regimen is feasible for this combined injury [22, 29]. However, due to the lack of documented data in the available literature regarding the timing of return to the previous activity level and, of course, the uniqueness of each possible combination of injury, the rehabilitation regimen should be decided on an individual basis.

### Injury mechanism

The most common mechanism of injury described is a sudden anterior translation of the tibia, which explains the rupture of the ACL, followed by a quadriceps contraction in a slightly flexed knee and fixed foot. The stress on the extensor mechanism and especially on the PT is greater in a partially flexed position, explaining the rupture of the PT [64]. The reported valgus stress in addition to rotational stress also explains why MCL rupture and medial or lateral meniscus rupture were the most common concomitant injuries.

### Anatomical location of the PT rupture

An interesting finding relates to the anatomical location of the PT rupture. Most studies described a mid-substance rupture [10, 14, 23, 24, 29–31, 33, 36–38, 47, 50, 53, 61]. Distal avulsion has been associated with a high-energy trauma, most notably demonstrated by Quinn et al. [49]. Therefore, the need to look for concomitant injuries in case of a distal PT rupture is even more important.

### Diagnostic and therapeutic algorithm

**Fig. 2:**
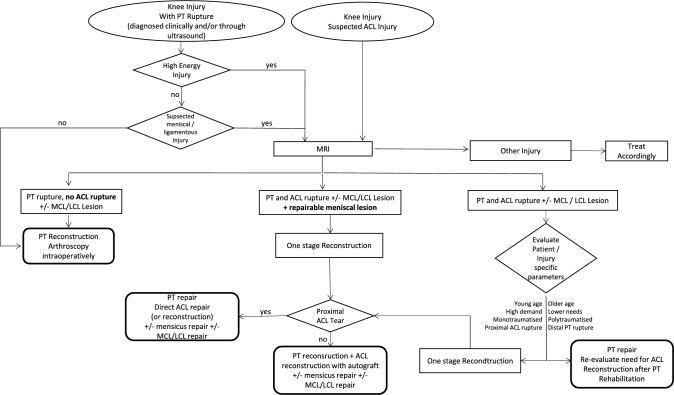
Diagnostic and therapeutic algorithm of simultaneus ACL and PT injuries based on the results of the systematic review and metaanalysis (see also Additional Files)

Taking into account all of the above, it is a challenge for the treating physician to make the correct decisions under time pressure. To assist, a diagnostic and therapeutic algorithm was developed based on the results of this review, summarizing all the points discussed above (Fig. 2).

### Strengths and limitations

The state-of-the-art review methodology, allowed for the inclusion of all available literature and the best possible data synthesis. However, this study has several limitations. First, the available literature is limited to case reports and case series with a maximum of six patients. The small sample size, consisting of only 56 patients limits the findings and their significance and may lead to an unknown selection bias due to the uneven quality and scope of the included studies. Secondly, despite efforts to synthesize available data comprehensively, the heterogeneity in study design and the data quality among the included studies posed challenges in achieving robust statistical significance. In addition, while our meta-analysis could provide valuable insights into the importance of MRI in the diagnosis of concomitant patella tendon and ACL ruptures, a significant concern is the possibility of publication bias because studies that report missed diagnoses are more likely to be published due to the perceived importance and clinical implications of these findings. Furthermore, the lack of documented data regarding the time of return to the previous activity level and the lack of postoperative or posttraumatic clinical and functional scores is an important drawback, meaning that conclusions should be drawn with caution. Finally, while the algorithm proposed in this study reflects the authors’ insights gained from a thorough analysis of the existing literature, its application remains contingent on individual surgeon preferences rather than being strictly evidence-based. Moreover, it is recognized that no algorithm could cover the full range of the possible concomitant injuries. Therefore decisions regarding management, especially for less common injuries, necessitate a personalized, case-by-case approach guided by both surgeon preference and patient-specific considerations.

## Conclusion

Combined ACL and PT rupture is a rare injury. Other concomitant injuries are common. Recognizing the full extent of this injury is key to successful management. It is important to perform an MRI in patients with PT rupture resulting from a high-energy trauma and a diagnostic arthroscopy / arthrotomy in cases where an MRI was not performed. PT ruptures should be treated surgically. For ACL rupture, conservative and operative treatment, one- or two-stage surgeries are possible depending on the patient’s profile and presence of other concomitant injuries. This systematic review provides a diagnostic and therapeutic algorithm to make the best possible, evidence-based decisions in this difficult injury setting.

## Conflict of interest

The authors declare no conflicts of interest.

## Ethical approval

Ethical review and approval were waived for this study because unlike primary research, no new personal, sensitive or confidential information was collected from participants. Only publicly available documents were used for the systematic review.

## Informed consent

Due to the nature of the study, no “Informed Consent” was necessary.

## Supplementary Information

Below is the link to the electronic supplementary material.

Supplementary file1 (DOCX 18 kb)

Supplementary file2 (DOCX 19 kb)

## References

[1] Achkoun A, Houjairi K, Quahtan O, Hassoun J, Arssi M, Rahmi M et al (2016) Simultaneous rupture of the anterior cruciate ligament and the patellar tendon: a case report. Pan Afr Med J 23:20 27366288 10.11604/pamj.2016.23.20.8621 PMC4916786

[2] Ansari S, Graham J (1995) Ipsilateral rupture of patellar tendon and anterior cruciate ligament: result of sports injury to the knee. Sports Exerc Injury 1:214–215

[3] Baker BE (1980) O’Donoghue’s triad plus patellar tendon rupture. N Y State J Med 80:1436–1437 6938816

[4] Bales CP, Guettler JH, Moorman CT 3rd (2004) Anterior cruciate ligament injuries in children with open physes: evolving strategies of treatment. Am J Sports Med 32:1978–1985 15572331 10.1177/0363546504271209

[5] Boublik M, Schlegel T, Koonce R, Genuario J, Lind C, Hamming D (2011) Patellar tendon ruptures in National Football League players. Am J Sports Med 39:2436–2440 21813442 10.1177/0363546511417083

[6] Bramer WM, Giustini D, de Jonge GB, Holland L, Bekhuis T (2016) De-duplication of database search results for systematic reviews in EndNote. J Med Libr Assoc 104:240–243 27366130 10.3163/1536-5050.104.3.014 PMC4915647

[7] Brunkhorst J, Johnson DL (2015) Multiligamentous knee injury concomitant with a patellar tendon rupture. Orthopedics 38:45–48 25611410 10.3928/01477447-20150105-06

[8] Buhl L, Muller S, Nuesch C, Pagenstert G, Mundermann A, Egloff C (2023) Functional leg performance 2 years after ACL surgery: a comparison between InternalBrace-augmented repair versus reconstruction versus healthy controls. J Orthop Traumatol 24:52 37735271 10.1186/s10195-023-00723-5 PMC10513977

[9] Capogna B, Strauss E, Konda S, Dayan A, Alaia M (2017) Distal patellar tendon avulsion in association with high-energy knee trauma: A case series and review of the literature. Knee 24:468–476 27916579 10.1016/j.knee.2016.10.020

[10] Chiang AS, Shin SS, Jazrawi LM, Rose DJ (2005) Simultaneous ipsilateral ruptures of the anterior cruciate ligament and patellar tendon: a case report. Bull Hosp Jt Dis 62:134–136 16022228

[11] Chiba K (2013) Surgical treatment of simultaneous rupture of the anterior cruciate ligament and the patellar tendon. J Knee Surg 26:S40–S44 23288727 10.1055/s-0031-1286195

[12] Chow FY, Wun YC, Chow YY (2006) Simultaneous rupture of the patellar tendon and the anterior cruciate ligament: a case report and literature review. Knee Surg Sports Traumatol Arthrosc 14:1017–1020 16489475 10.1007/s00167-006-0048-3

[13] Clayton RA, Court-Brown CM (2008) The epidemiology of musculoskeletal tendinous and ligamentous injuries. Injury 39:1338–1344 19036362 10.1016/j.injury.2008.06.021

[14] Costa-Paz M, Muscolo DL, Makino A, Ayerza MA (2005) Simultaneous acute rupture of the patellar tendon and the anterior cruciate ligament. Arthroscopy 21:1143 16171641 10.1016/j.arthro.2005.05.028

[15] Cucchi D, Aliprandi A, Nocerino E, Randelli P (2018) Early combined arthroscopic treatment for simultaneous ruptures of the patellar tendon and the anterior cruciate ligament leads to good radiological results and patient satisfaction. Knee Surg Sports Traumatol Arthrosc 26:1164–1173 28456816 10.1007/s00167-017-4562-2

[16] De Rousiers A, Barbier O, Bouchard A, Nguyen R, Choufani C (2020) Simultaneous rupture of the patellar, anterior cruciate and medial collateral ligaments: analysis of a case and exhaustive review of the literature. J Traumatol Sport 37:162–165

[17] Diermeier T, Rothrauff BB, Engebretsen L, Lynch AD, Ayeni OR, Paterno MV et al (2020) Treatment after anterior cruciate ligament injury: panther symposium ACL treatment consensus group. Knee Surg Sports Traumatol Arthrosc 28:2390–2402 32388664 10.1007/s00167-020-06012-6 PMC7524809

[18] Domnick C, Garcia P, Raschke MJ et al (2017) Trends and incidences of ligament-surgeries and osteotomies of the knee: an analysis of German inpatient records 2005–2013. Arch Orthop Trauma Surg 137(7):989–995 28466182 10.1007/s00402-017-2704-0

[19] Enad JG (1999) Patellar tendon ruptures. South Med J 92:563–566 10372848 10.1097/00007611-199906000-00003

[20] Fitzgerald GK, Axe MJ, Snyder-Mackler L (2000) A decision-making scheme for returning patients to high-level activity with nonoperative treatment after anterior cruciate ligament rupture. Knee Surg Sports Traumatol Arthrosc 8:76–82 10795668 10.1007/s001670050190

[21] Fox MA, Engler ID, Zsidai BT, Hughes JD, Musahl V (2023) Anatomic anterior cruciate ligament reconstruction: Freddie Fu’s paradigm. J ISAKOS 8:15–22 35988888 10.1016/j.jisako.2022.08.003

[22] Frosch KH, Preiss A, Heider S, Stengel D, Wohlmuth P, Hoffmann MF et al (2013) Primary ligament sutures as a treatment option of knee dislocations: a meta-analysis. Knee Surg Sports Traumatol Arthrosc 21:1502–1509 22868350 10.1007/s00167-012-2154-8 PMC3685709

[23] Futch LA, Garth WP, Folsom GJ, Ogard WK (2007) Acute rupture of the anterior cruciate ligament and patellar tendon in a collegiate athlete. Arthroscopy 23(112):e111-114 10.1016/j.arthro.2005.07.030 17210443

[24] Gülabi D, Erdem M, Bulut G, Sağlam F (2014) Neglected patellar tendon rupture with anterior cruciate ligament rupture and medial collateral ligament partial rupture. Acta Orthop Traumatol Turc 48:231–235 24747636 10.3944/AOTT.2014.3149

[25] Haddaway NR, Grainger MJ, Gray CT (2022) Citationchaser: a tool for transparent and efficient forward and backward citation chasing in systematic searching. Res Synth Methods 13:533–545 35472127 10.1002/jrsm.1563

[26] Hoogeslag RAG, Huis In’t Veld R, Brouwer RW, de Graaff F, Verdonschot N (2022) Acute anterior cruciate ligament rupture: repair or reconstruction? Five-year results of a randomized controlled clinical trial. Am J Sports Med 50:1779–1787 35486517 10.1177/03635465221090527

[27] Hsu H, Siwiec RM (2023) Patellar tendon rupture. StatPearls. Treasure Island (FL) 30020647

[28] Iriuchishima T, Horaguchi T, Morimoto Y et al (2010) Intensity of physiotherapy after anterior cruciate ligament reconstruction: a comparison of two rehabilitation regimen. Arch Orthop Trauma Surg 130(8):1053–1058 20559646 10.1007/s00402-010-1134-z

[29] Ismailidis P, Kernen R, Egloff C, Nuesch C, Mundermann A, Muller SA (2020) Clinical and biomechanical outcomes of one-stage treatment of a simultaneous ipsilateral patellar tendon and ACL tear combined with a Tibial Plateau fracture: a case study. Case Rep Orthop 2020:5793948 32089930 10.1155/2020/5793948 PMC7024102

[30] Kim DH, Lee GC, Park SH (2014) Acute simultaneous ruptures of the anterior cruciate ligament and patellar tendon. Knee Surg Relat Res 26:56–60 24639949 10.5792/ksrr.2014.26.1.56 PMC3953527

[31] Koukoulias NE, Koumis P, Papadopoulos A, Kyparlis D, Papastergiou SG (2011) Acute, simultaneous tear of patellar tendon and ACL: possible mechanism of injury and rationality of the two-stage surgical treatment. BMJ Case Rep. 10.1136/bcr.05.2011.4178 22687668 10.1136/bcr.05.2011.4178 PMC4545034

[32] Lai CCH, Ardern CL, Feller JA, Webster KE (2018) Eighty-three per cent of elite athletes return to preinjury sport after anterior cruciate ligament reconstruction: a systematic review with meta-analysis of return to sport rates, graft rupture rates and performance outcomes. Br J Sports Med 52:128–138 28223305 10.1136/bjsports-2016-096836

[33] Levakos Y, Sherman MF, Shelbourne KD, Trakru S, Bonamo JR (1996) Simultaneous rupture of the anterior cruciate ligament and the patellar tendon. Six case reports. Am J Sports Med 24:498–503 8827310 10.1177/036354659602400415

[34] Levy BA, Krych AJ, Shah JP, Morgan JA, Stuart MJ (2010) Staged protocol for initial management of the dislocated knee. Knee Surg Sports Traumatol Arthrosc 18:1630–1637 20635077 10.1007/s00167-010-1209-y

[35] Li LZ, Deng XT, Li Z, Liu JC (2022) Single-staged arthroscopic treatment of simultaneous ruptures of the anterior cruciate ligament and the patellar tendon: a case report. Asian J Surg 45:1942–1943 35443930 10.1016/j.asjsur.2022.04.020

[36] Lobo JO, Cherian JJ, Sahu A (2017) Case of Acute concomitant rupture of anterior cruciate ligament and patellar tendon of knee: surgical decision making and outcome. J Orthop Case Rep 7:5–8 29051869 10.13107/jocr.2250-0685.780 PMC5635187

[37] Mariani PP, Cerullo G, Iannella G (2013) Simultaneous rupture of the patellar tendon and the anterior cruciate ligament: report of three cases. J Knee Surg 26(Suppl 1):S53-57 23288778 10.1055/s-0031-1299653

[38] Matava MJ (1996) Patellar tendon ruptures. J Am Acad Orthop Surg 4:287–296 10797196 10.5435/00124635-199611000-00001

[39] Matthews AH, Fraser EJ, Parkinson B (2018) Management of simultaneous patellar tendon and anterior cruciate ligament ruptures-a systematic review of available literature. J Orthop Trauma 32:e320–e326 29782440 10.1097/BOT.0000000000001219

[40] McCormack RG, Dryden PJ (1998) Simultaneous rupture of the anterior cruciate ligament and patellar tendon. Clin J Sport Med 8:307–309 9884796 10.1097/00042752-199810000-00009

[41] McKinney B, Cherney S, Penna J (2008) Intra-articular knee injuries in patients with knee extensor mechanism ruptures. Knee Surg Sports Traumatol Arthrosc 16:633–638 18478204 10.1007/s00167-008-0516-z

[42] Meheux CJ, Jack RA 2nd, McCulloch PC, Lintner DM, Harris JD (2018) Surgical management of simultaneous anterior cruciate ligament and patellar tendon ruptures: a systematic review. J Knee Surg 31:875–883 29284175 10.1055/s-0037-1615814

[43] Murray MM (2021) Optimizing outcomes of ACL surgery-Is autograft reconstruction the only reasonable option? J Orthop Res 39:1843–1850 34191344 10.1002/jor.25128 PMC8387392

[44] Nebelung W, Wuschech H (2005) Thirty-five years of follow-up of anterior cruciate ligament-deficient knees in high-level athletes. Arthroscopy 21:696–702 15944625 10.1016/j.arthro.2005.03.010

[45] Page MJ, McKenzie JE, Bossuyt PM, Boutron I, Hoffmann TC, Mulrow CD et al (2021) The PRISMA 2020 statement: an updated guideline for reporting systematic reviews. BMJ 372:n71 33782057 10.1136/bmj.n71 PMC8005924

[46] Pérez J, Novoa GA, Pierobon A, Soliño S, Calvo Delfino M, Sajfar ME et al (2018) Postoperative rehabilitation of simultaneous rupture of anterior cruciate ligament and patellar ligament: a case report. Physiother Res Int J Res Clin Phys Ther 23:e1735 10.1002/pri.1735 30058203

[47] Perwanger F, Jeske HC (2020) Kombinierte Ruptur des vorderen Kreuzbandes und des Ligamentum patellae mit inkarzerierter Außenmeniskuskorbhenkelruptur. Knie Journal 2:297–301

[48] Pointinger H, Munk P, Poeschl GP (1999) Rupture of superficial pes anserinus and partial rupture of patellar ligament as rare concomitant lesions of complex knee joint injuries. Unfallchirurg 102:69–73 10095409 10.1007/s001130050374

[49] Quinn M, Lemme N, Anna King DO, Tabaddor RR (2019) Simultaneous rupture of the anterior cruciate ligament, medial collateral ligament and patellar tendon: a case series, review of the literature, and proposed treatment algorithm. Int J Sports Exerc Med 5:144. 10.23937/2469-5718/1510144

[50] Rae PJ, Davies DR (1991) Simultaneous rupture of the ligamentum patellae, medial collateral, and anterior cruciate ligaments. A case report. Am J Sports Med 19:529–530 1741474 10.1177/036354659101900522

[51] Rahardja R, Love H, Clatworthy MG, Young SW (2023) Meniscal repair failure following concurrent primary anterior cruciate ligament reconstruction: results from the New Zealand ACL Registry. Knee Surg Sports Traumatol Arthrosc. 10.1007/s00167-023-07424-w 37145132 10.1007/s00167-023-07424-w PMC10471701

[52] Sanders TL, Maradit Kremers H, Bryan AJ, Larson DR, Dahm DL, Levy BA et al (2016) Incidence of anterior cruciate ligament tears and reconstruction: a 21-year population-based study. Am J Sports Med 44:1502–1507 26920430 10.1177/0363546516629944

[53] Schmidt-Wiethoff R, Tüylü H, Dargel J, Schneider T (2003) Simultaneous rupture of the patellar tendon and the anterior cruciate ligament. Aktuelle Traumatol 33:35–38

[54] Selva-Sarzo F, Nebot-Sanchis LI (2022) Combined rupture of the right anterior cruciate ligament and patellar tendon on a 28-year-old spanish professional handball player successfully treated by single-stage reconstructive surgery. Am J Case Rep. 10.12659/AJCR.937689 36346777 10.12659/AJCR.937689 PMC9662075

[55] Shillington M, Logan M, Watts M, Myers P (2008) A complex knee injury in a rugby league player. combined rupture of the patellar tendon, anterior cruciate and medial collateral ligaments, with a medial meniscal tear. Injury Extra 39:327–328

[56] Tsarouhas A, Iosifidis M, Kotzamitelos D, Traios S (2011) Combined rupture of the patellar tendon, anterior cruciate ligament and lateral. Hippokratia 15:178–180 22110304 PMC3209685

[57] van der List JP, Mintz DN, DiFelice GS (2017) The location of anterior cruciate ligament tears: a prevalence study using magnetic resonance imaging. Orthop J Sports Med 5:2325967117709966 28680889 10.1177/2325967117709966 PMC5484434

[58] van Melick N, van Cingel RE, Brooijmans F, Neeter C, van Tienen T, Hullegie W et al (2016) Evidence-based clinical practice update: practice guidelines for anterior cruciate ligament rehabilitation based on a systematic review and multidisciplinary consensus. Br J Sports Med 50:1506–1515 27539507 10.1136/bjsports-2015-095898

[59] Vega R, Huanquilef L, Íñiguez M (2006) Rotura simultánea aguda del tendón patelar y ligamento cruzado anterior. Rev Chilena Ortop y Traum. 47:131–134 (**Google Scholar**)

[60] Verma N, Singh H, Mohammad N, Srivastav S (2018) Concomitant multiligamentous knee injury and patellar tendon tear- a rare injury pattern. J Arthrosc Joint Surg 5:183–186

[61] Wissman RD, Vonfischer N, Kempf K (2012) Acute concomitant anterior cruciate ligament and patellar tendon tears in a non-dislocated knee. J Clin Imaging Sci 2:3 22439127 10.4103/2156-7514.93035 PMC3307214

[62] Xie T, Han X, Zhou SB, Zhu LL, He QF (2021) A case report of multi-ligaments injury of the ACL-MCL-PT combined with an occult fracture of the posterolateral tibial plateau. Trauma Case Rep 33:100457 33855154 10.1016/j.tcr.2021.100457 PMC8025053

[63] Yaari L, Singer J, Goldberg D et al (2024) Eighteen-year outcome of anterior cruciate ligament reconstruction with patellar tendon or hamstring autograft. Arch Orthop Trauma Surg 144(5):2189–2195 38630253 10.1007/s00402-024-05317-2

[64] Zernicke RF, Garhammer J, Jobe FW (1977) Human patellar-tendon rupture. J Bone Joint Surg Am 59:179–183 845201

