# Supplementary material for: Simultaneous patellar tendon and anterior cruciate ligament rupture: a systematic review, meta-analysis and algorithmic approach
Source: Arch Orthop Trauma Surg. 2024 Dec 18;145(1):61. doi: 10.1007/s00402-024-05676-w (PMC11655589; doi:10.1007/s00402-024-05676-w)
Supplement: Supplementary file 1 — Supplementary file1 (DOCX 18 kb) [file 402_2024_5676_MOESM1_ESM.docx]

# Appendices

# Appendix 1: Search strategies

## Embase.com

('patellar ligament'/de OR 'patellar tendon rupture'/de OR 'patella tendon rupture'/de OR ((patellar OR patella OR patellae) NEAR/3 (tendon* OR ligament*)):ab,ti)

AND

('anterior cruciate ligament'/de OR 'knee cruciate ligament'/de OR 'anterior cruciate ligament injury'/exp OR ((('anterior cruciate' OR 'anterior crucial') NEAR/4 ligament*) OR ACL OR 'O Donoghue s triad' OR 'unhappy triad'):ab,ti)

AND

('anterior cruciate ligament injury'/exp OR 'patellar tendon rupture'/de OR 'patella tendon rupture'/de OR 'sport injury'/de OR 'knee ligament injury'/de OR 'knee injury'/de OR 'tendon rupture'/de OR 'rupture'/de OR 'injury'/de OR (rupture* OR tear OR tears OR tore OR torn OR injury OR injuries OR injured OR rip* OR wreng* OR wrest*):ab,ti)

NOT

(('animal'/de OR 'animal experiment'/exp OR 'nonhuman'/de) NOT ('human'/exp OR 'human experiment'/de))

NOT

[conference abstract]/lim

### Ovid Medline

(patellar ligament/ OR ((patellar OR patella OR patellae) ADJ3 (tendon* OR ligament*)).ab,ti.)

AND

(anterior cruciate ligament/ OR anterior cruciate ligament injuries/ OR (((anterior cruciate OR anterior crucial) ADJ4 ligament*) OR ACL OR "O'Donoghue's triad" OR unhappy triad).ab,ti.)

AND

(anterior cruciate ligament injuries/ OR athletic injuries/ OR knee injuries/ OR tendon injuries/ OR rupture/ OR injuries.fs. OR (rupture* OR tear OR tears OR tore OR torn OR injury OR injuries OR injured OR rip* OR wreng* OR wrest*).ab,ti.)

NOT

(exp animals/ NOT humans/)

## Scopus

TITLE-ABS(((patellar OR patella OR patellae) W/2 (tendon* OR ligament*)) AND ((("anterior cruciate" OR "anterior crucial") W/3 ligament*) OR ACL OR "O'Donoghue's triad" OR "unhappy triad") AND (rupture* OR tear OR tears OR tore OR torn OR injury OR injuries OR injured OR rip* OR wreng* OR wrest*))

## SportDiscus

((DE "patellar ligament") OR (DE "patellar ligament injuries") OR ((TI patellar OR AB patellar OR TI patella OR AB patella OR TI patellae OR AB patellae) AND (TI tendon* OR AB tendon* OR TI ligament* OR AB ligament*)))

AND

((DE "anterior cruciate ligament") OR (DE "anterior cruciate ligament injuries") OR (((TI "anterior cruciate" OR AB "anterior cruciate" OR TI "anterior crucial" OR AB "anterior crucial") AND (TI ligament* OR AB ligament*)) OR TI ACL OR AB ACL OR TI "O'Donoghue's triad" OR AB "O'Donoghue's triad" OR TI "unhappy triad" OR AB "unhappy triad"))

AND

((DE "anterior cruciate ligament injuries") OR (DE "SPORTS injuries" OR DE "AEROBICS injuries" OR DE "AQUATIC sports injuries" OR DE "BASEBALL injuries" OR DE "BASKETBALL injuries" OR DE "BOXING injuries" OR DE "COMMOTIO cordis" OR DE "CRICKET injuries" OR DE "EQUESTRIAN accidents" OR DE "FOOTBALL injuries" OR DE "GOLF injuries" OR DE "GYMNASTICS injuries" OR DE "HIKING injuries" OR DE "HOCKEY injuries" OR DE "HORSE sports injuries" OR DE "IN-line skating injuries" OR DE "JOGGING injuries" OR DE "JUDO injuries" OR DE "JUMPER'S knee" OR DE "KARATE injuries" OR DE "MARTIAL arts injuries" OR DE "MOTORSPORTS injuries" OR DE "NETBALL injuries" OR DE "RACKET game injuries" OR DE "RUGBY football injuries" OR DE "RUNNING injuries" OR DE "SKATEBOARDING injuries" OR DE "SOCCER injuries" OR DE "TENNIS injuries" OR DE "VAULTING injuries" OR DE "VOLLEYBALL injuries" OR DE "WALKING (Sports) injuries" OR DE "WEIGHT training injuries" OR DE "WINTER sports injuries") OR (DE "SPORTS injuries in children") OR (DE "knee injuries") OR (DE "tendon injuries") OR (DE "patellar ligament injuries") OR (TI rupture* OR AB rupture* OR TI tear OR AB tear OR TI tears OR AB tears OR TI tore OR AB tore OR TI torn OR AB torn OR TI injury OR AB injury OR TI injuries OR AB injuries OR TI injured OR AB injured OR TI rip* OR AB rip* OR TI wreng* OR AB wreng* OR TI wrest* OR AB wrest*))
