# Supplementary material for: Simultaneous patellar tendon and anterior cruciate ligament rupture: a systematic review, meta-analysis and algorithmic approach
Source: Arch Orthop Trauma Surg. 2024 Dec 18;145(1):61. doi: 10.1007/s00402-024-05676-w (PMC11655589; doi:10.1007/s00402-024-05676-w)
Supplement: Supplementary file 2 — Supplementary file2 (DOCX 19 kb) [file 402_2024_5676_MOESM2_ESM.docx]

Supplementary material 2: Assessment of the methodological quality of all included studies according to the Joanna Briggs Institute (JBI) Critical Appraisal Checklist and risk of bias for case series and case reports.

**Case Series**

| Author  (year) | **Were there clear criteria for inclusion in the case series?** | **Was the condition measured in a standard, reliable way for all participants included in the case series?** | **Were valid methods used for identification of the condition for all participants included in the case series?** | **Did the case series have consecutive inclusion of participants?** | **Did the case series have complete inclusion of participants?** | **Was there clear reporting of the demographics of the participants in the study?** | **Was there clear reporting of clinical information of the participants?** | **Were the outcomes or follow-up results of cases clearly reported?** | **Was there clear reporting of the presenting site(s)/clinic(s) demographic information?** | **Was statistical analysis appropriate?** |
| --- | --- | --- | --- | --- | --- | --- | --- | --- | --- | --- |
| Costa -Paz (2005)  [14] | No | Yes | Yes | No | No | Yes | Yes | Yes | Not applicable | Not applicable |
| Levakos (1996)  [31] | No | Yes | No | No | No | Yes | Yes | No | Not applicable | Not applicable |
| Mariani (2013) [35] | No | Yes | Yes | No | No | Yes | Yes | Yes | Not applicable | Not applicable |
| Capogna (2017)  [9] | No | Yes | No | Yes | Yes | Yes | Yes | No | Not applicable | Not applicable |
| Quinn (2019) [45] | No | Yes | Yes | No | No | Yes | Yes | Yes | Not applicableq | Not applicable |
| Boublik (2011) [5] | No | Yes | Yes | Yes | Yes | Yes | No | No | Yes | Not applicable |

**Case reports**

| Author (year) | **Were patient’s demographic characteristics clearly described?** | **Was the patient’s history clearly described and presented as a timeline?** | **Was the current clinical condition of the patient on presentation clearly described?** | **Were diagnostic tests or methods and the results clearly described?** | **Was the intervention(s) or treatment procedure(s) clearly described?** | **Was the post-intervention clinical condition clearly described?** | **Were adverse events (harms) or unanticipated events identified and described?** | **Does the case report provide takeaway lessons?** |
| --- | --- | --- | --- | --- | --- | --- | --- | --- |
| Baker (1980) [3] | Yes | Yes | Yes | No | Yes | Yes | Not applicable | Yes |
| Brunkhorst (2015) [7] | Yes | No | Yes | Yes | Yes | No | Not applicable | No |
| Chiang (2005) [10] | Yes | No | Yes | Yes | Yes | Yes | Yes | Yes |
| Chiba (2013) [11] | Yes | No | Yes | Yes | Yes | Yes | Not applicable | Yes |
| Chow (2006) [12] | Yes | Yes | Yes | No | Yes | Yes | Yes | Yes |
| Cucchi (2017) [15] | Yes | No | Yes | Yes | Yes | Yes | Yes | Yes |
| Cucchi (2017) [15] | Yes | No | Yes | Yes | Yes | Yes | Yes | Yes |
| Futch (2007) [22] | Yes | No | Yes | Yes | Yes | Yes | Yes | Yes |
| Kim (2014) [28] | Yes | No | Yes | Yes | Yes | Yes | Yes | Yes |
| Koukoulias (2011) [29] | Yes | Yes | Yes | No | Yes | Yes | Yes | Yes |
| Mccormack (1998) [38] | Yes | Yes | Yes | No | Yes | No | Not applicable | Yes |
| Rae (1991) [48] | Yes | No | Yes | No | Yes | Yes | Not applicable | Yes |
| Shillington (2008) [53] | Yes | No | Yes | Yes | Yes | No | Yes | Yes |
| Tsarouhas (2011) [54] | Yes | No | Yes | No | Yes | Yes | Not applicable | Yes |
| Wissman (2012) [59] | Yes | No | Yes | Yes | Yes | Yes | Yes | Yes |
| Gülabi (2014) [23] | Yes | No | Yes | Yes | Yes | Yes | Yes | Yes |
| Achkoun (2016) [1] | Yes | Yes | Yes | Yes | Yes | Yes | Not applicable | Yes |
| Schmidt-Wiethoff (2003) [51] | Yes | No | Yes | Yes | Yes | Yes | Not applicable | Yes |
| Vega (2006) [57] | Yes | Yes | Yes | Yes | Yes | Yes | Not applicable | Yes |
| Perez (2018) [44] | Yes | Yes | Yes | Yes | Yes | Yes | Not applicable | Yes |
| Mathews (2018) [37] | Yes | No | Yes | Yes | Yes | Yes | Not applicable | Yes |
| Lobo (2017) [34] | Yes | No | Yes | Yes | Yes | Yes | Not applicable | Yes |
| Ismailidis (2019) [27] | Yes | Yes | Yes | No | Yes | Yes | Yes | Yes |
| Verma (2018) [58] | Yes | No | Yes | Yes | Yes | Yes | Not applicable | Yes |
| Verma (2018) [58] | Yes | No | Yes | Yes | No | Yes | Not applicable | Yes |
| Ansari (1995) [2] | Yes | No | No | No | No | Yes | Yes | Yes |
| Pointinger (1999) [46] | Yes | No | Yes | No | Yes | Yes | Not applicable | Yes |
| Perwanger (2020) [45] | Yes | No | Yes | Yes | Yes | Yes | Not applicable | Yes |
| Xie (2021) [60] | Yes | No | Yes | Yes | Yes | Yes | Not applicable | Yes |
| A. De Rousiers (2020) [16] | Yes | Yes | Yes | Yes | Yes | Yes | Not applicable | Yes |
| Selva-Sarzo (2022) [52] | Yes | No | Yes | Yes | Yes | Yes | Yes | Yes |
| Zhong Li (2022) [33] | Yes | No | Yes | Yes | Yes | Yes | Not applicable | Yes |
